# Office-Based, Single-Sided, Low-Field MRI-Guided Prostate Biopsy

**DOI:** 10.7759/cureus.25021

**Published:** 2022-05-15

**Authors:** Poorvi Satya, John Adams Jr., Srirama S Venkataraman, Dinesh Kumar, Ram Narayanan, Aleksandar Nacev, Joseph N Macaluso Jr.

**Affiliations:** 1 Operations, Mailman School of Public Health, Columbia University, New York, USA; 2 Urology, Mississippi Urology Clinic, PLLC., Jackson, USA; 3 Operations, Promaxo Inc., Oakland, USA; 4 Engineering, Promaxo Inc., Oakland, USA; 5 Urology, Louisiana State University Health Foundation, New Orleans, USA

**Keywords:** prostate biopsy, mri, urology, male urology, mr-guided biopsy, prostate cancer, office-based mri, low-field mri, targeted prostate biopsy

## Abstract

This paper describes the workflow of transperineal prostate biopsy (TBx) using the single-sided, low-field Promaxo MRI system (Promaxo Inc., Oakland, California, United States) operating at a field strength ranging between 58 and 74 millitesla (mT). Prostate cancer (PCa) is the leading cause of cancer-related death and the second most frequently diagnosed cancer in men. Systematic biopsy (SBx) with 12-14 cores is the preferred standard of care procedure. The blinded approach of SBx, however, results in several shortcomings, including high rates of false negatives and increased infection rates due to the transrectal approach. The evolution of clinical use and scientific research using different prostate biopsy modalities is discussed, including the potential for the Promaxo MRI system to mitigate logistical constraints often associated with standard magnetic resonance (MR)-guided biopsy through the utilization of an office-based, low-field MRI.

## Introduction

Prostate cancer (PCa) is the second most frequently diagnosed cancer in men. Early-stage PCa is asymptomatic and may have an indolent course. Men with a family history of PCa and men of African American descent are disproportionately affected, placing them at greater risk of the disease [1]. Systematic biopsy (SBx) with transrectal ultrasound (TRUS) is the current preferred office-based procedure due to its speed and the limited footprint the ultrasound system requires [2]. Fusion biopsies (FBx), where a pre-procedure diagnostic MRI is fused with real-time TRUS to guide biopsies, have been in use for more than a decade, offering urologists an alternative that allows for more precise targeting of specific lesions, with a prostate imaging reporting and data system (PI-RADS) score greater than three. The variance in registration of pre-procedure MRI in combination with real-time ultrasound, however, can suffer due to prostate gland deformation from the TRUS probe. This, along with the steep learning curve of mapping disparate modalities, has limited the wider adoption of FBx [3,4]. Additionally, a transrectal biopsy can result in infections, sometimes serious and requiring hospitalization. Bothersome side-effects of transrectal biopsy also include hematuria and hematospermia. Rarely, significant rectal bleeding can occur [5]. Direct in-bore MRI-guided biopsies are also performed, which results in higher cancer detection rates; however, the logistics, duration, and costs associated with the procedure limit its use to a few academic medical centers [6,7]. We report the technical workflow of a novel point-of-care low-field MRI system clinical workflow for performing TBx within an office setting at a standard outpatient clinic.

## Technical report

**Figure 1:**
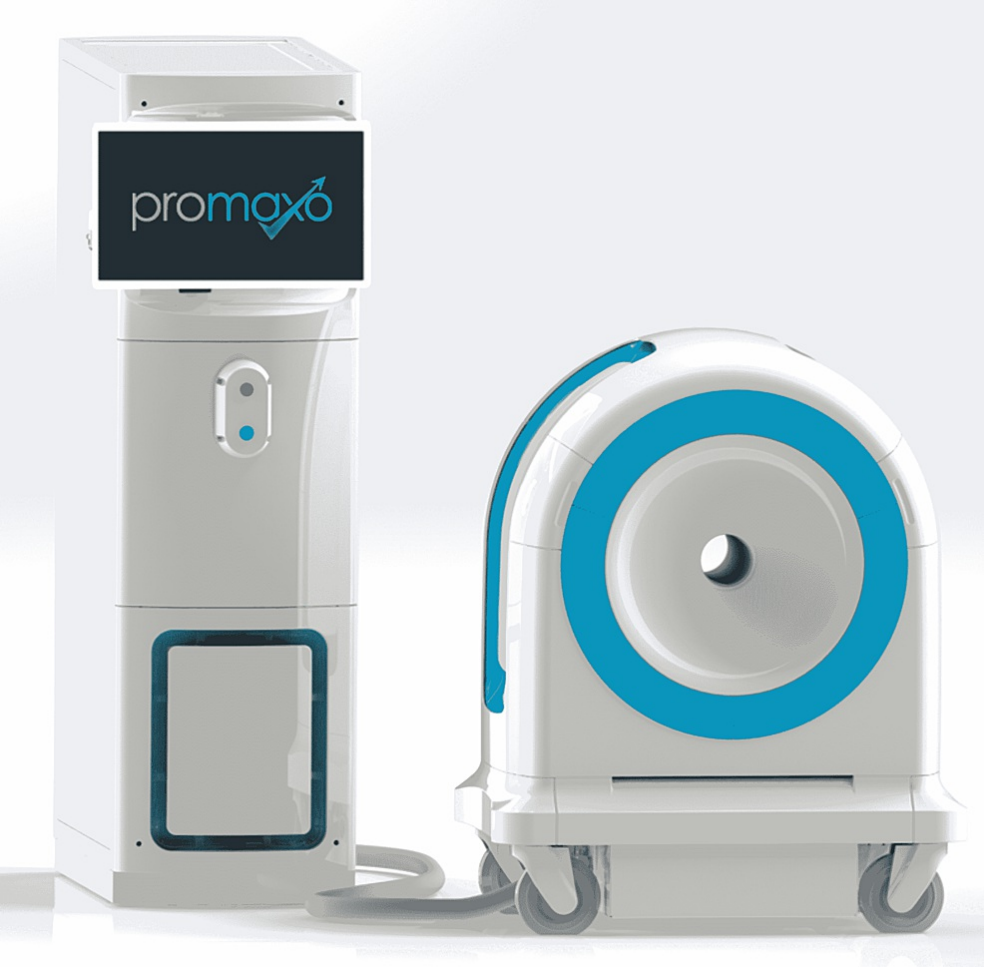
The Promaxo MRI system consisting of the magnet (ranging from 58mT to 74mT) and accompanying electronic rack with an attached graphical user interface (GUI). Promaxo Inc., Oakland, California, United States

Patients with a finding of an elevated prostate-specific antigen (PSA) and/or suspicious digital rectal examination (DRE) are referred for prostate biopsy. The initial study site is Mississippi Urology Clinic, PLLC., Jackson, Mississippi, United States. Patients undergo a multi-parametric MRI (mpMRI) on a commercial whole-body 3 Tesla (T) MRI (Philips Healthcare, Amsterdam, The Netherlands) prior to the biopsy, as per PI-RADS version two protocol. A board-certified radiologist identifies suspicious lesions. A PI-RADS assessment is assigned to the lesion and delineated (width, height, and depth, respectively). The patient then undergoes a transperineal biopsy (TBx) using the single-sided, low-field Promaxo MRI system (Promaxo Inc., Oakland, California, United States) with a field strength of 58-74mT. Patients who have magnetic resonance (MR)-sensitive pacemakers, implants, or other contraindications to MRI were excluded from the study. A pictorial representation of the Promaxo MRI system with the open-face configuration is shown in Figure 1.

The Promaxo MRI biopsy workflow consists of two parts. The first part is prior to the scheduled biopsy procedure, where a board-certified radiologist uses a digital imaging and communications in medicine (DICOM) viewer (Promaxo Inc., Oakland, California) to annotate the regions of interest on T2-weighted (T2W) 3T MRI scans of the patient obtained prior to the biopsy date. The annotated T2W images are then uploaded to the Promaxo MRI system. The second part entails the day of the procedure when the patient is placed in a high-lithotomy position and the pelvic region is enclosed within a five-channel surface coil with an additional single channel dedicated receive coil with MRI-visible fiducials that have an MR-visible biopsy grid for transperineal access. The patient’s pelvic region is positioned as close to the center of the field of view of the single-sided MR sufficient to image the entire prostate gland. 

The TBx procedure with Promaxo MRI included a board-certified urologist (JA) trained in the use of the Promaxo MRI system to register the imported 3T images with the acquired Promaxo MRI T2W scan of the subject. With the physical template coordinates and depth for each target displayed on the registered images, the urologist selects target locations, inserts the needle(s) transperineally through the template in the appropriate coordinate location and depth, and extracts tissue samples from the identified lesions.

**Figure 2:**
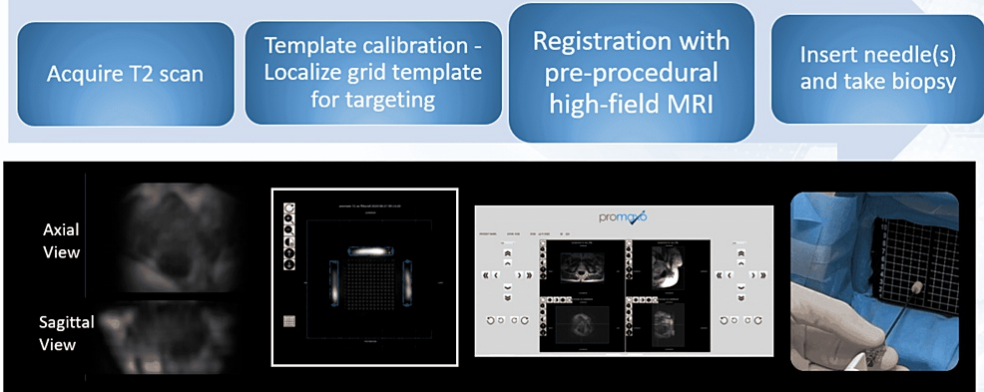
A typical clinical workflow when performing needle-guided intervention using the Promaxo MRI system. The user navigates through the various stages using the Promaxo graphical user interface (GUI). Promaxo Inc., Oakland, California, United States

An average of three cores are collected with the Promaxo MR-targeted method. Additionally, systematic biopsies or selected random biopsies are added to the targeted total as felt clinically indicated. A 20cm biopsy gun with an 18G biopsy needle (Max-Core™ Disposable Core Biopsy Instrument, C. R. Bard, Inc., Murray Hill, New Jersey, United States) and a 17G cannula (TruGuide™ Disposable Coaxial Biopsy Needle, C. R. Bard, Inc., Murray Hill, New Jersey, United States) is used to acquire cores in the Promaxo MRI-TBx procedure. Extracted biopsy samples are sent for pathologic analysis. The full clinical workflow is outlined in Figure 2.

## Discussion

SBx is the preferred and most common standard of care outpatient procedure at urologic clinics due to its ease of use and speed [8]. The random approach, however, results in a higher rate of false negatives [8]. The reported false-negative rates in SBx range from 30% to 60%. This technique does not detect around 30% of significant prostate cancers while upgrading or upstaging cancer after a positive TRUS confirmatory biopsy ranges between 25% to 40% [9,10]. In addition to underperformance in the detection and upgrading of cancers, higher infection rates are reported with patients undergoing transrectal as compared to transperineal biopsy procedures [9]. Prior studies have demonstrated the benefits of pre-biopsy MRI and MRI-guided targeted biopsies over blinded SBx [8,11,12]. In a recent study, Jayadevan et al. found the upstaging of cancer with FBx to be better than SBx [13]. TBx which uses mpMRI fusion with TRUS is gaining popularity in clinical practice over SBx, with the prognostic value highlighted in a recent study with 332 patients [10]. Although FBx is better than a systematic 12-core biopsy, it still has issues such as errors in fusing pre-procedure MRI with real-time ultrasound, and a steep learning curve [5,13]. In the case of the Promaxo MRI System, the pre-procedure 3T mpMRI with annotations has helped limit the number of cores to three, focusing on the targets that were found to be suspicious. Additionally, the mpMRI was used for the first time to guide the needle under, intra-procedure, low-field MRI. Since the co-registration is between images from the same modality (high and low-field MRI), the learning curve to localize and target the lesion should not be as steep as that between MRI and ultrasound in a fusion biopsies [14,15]. The co-registration of the T2W images obtained in the same axial orientation from both 3T and Promaxo resulted in lower navigation and registration errors making the MR-MR TBx more accurate in localizing lesions of interest [16]. The transperineal approach limits the potential infection often associated with transrectal procedures [17]. Additionally, the technology has an open-facing, quiet configuration and the absence of a required endorectal coil or other transrectal probes, which mitigates patient anxiety associated with claustrophobia and discomfort. Preliminary results from the initial study at Mississippi Urology Clinic also demonstrate the advantage of the system in detecting higher rates of clinically significant cancers as compared to SBx.

## Conclusions

A TBx approach can benefit patients significantly by favorably impacting the care pathway, providing as much as 6-12 months head-start in a definitive diagnosis and initiation of management. The technology demonstrates the possibility of now being able to conduct an MR-guided procedure within a standard outpatient clinic, resulting in a cancer detection rate that is equal to in-bore MRI procedures, while mitigating the logistical and cost constraints often associated with standard high field strength MRI systems. For the patient, potential post-procedural infection due to the transperineal approach is limited, and patient discomfort is eased with the absence of an endorectal coil or transrectal probe. The quiet, open-facing configuration of the system also decreases claustrophobia. Future studies are needed to compare MR-MR with MR-US FBx and to further understand the potential benefit of MR-MR over MR-US fusion.
